# Advances on the anti-tumor mechanisms of the carotenoid Crocin

**DOI:** 10.7717/peerj.15535

**Published:** 2023-06-29

**Authors:** Xingxun Bao, Jinhua Hu, Yan Zhao, Ruixue Jia, Hairong Zhang, Lei Xia

**Affiliations:** 1 Shandong University of Traditional Chinese Medicine, Jinan, China; 2 Shandong Provincial Hospital, Jinan, China; 3 The Third Hospital of Jinan, Jinan, China; 4 Shandong Provincial Third Hospital, Jinan, China

**Keywords:** Crocin, Tumor, Cancer, Nanoliposome, Mechanism

## Abstract

Saffron is located in the upper part of the crocus stigma of iridaceae, which has a long history of medicinal use. Crocin (molecular formula C_44_H_64_O_24_) is a natural floral glycoside ester compound extracted from saffron, which is a type carotenoid. Modern pharmacological studies have shown that crocin has multiple therapeutic effects including anti-inflammatory, anti-oxidant, anti-hyperlipidemic and anti-stone effects. In recent years, crocin has been widely noticed due to its considerable anti-tumor effects manifested by the induction of tumor cell apoptosis, inhibition of tumor cell proliferation, inhibition of tumor cell invasion and metastasis, enhancement of chemotherapy sensitivity and improvement of immune status. The anti-tumor effects have been shown in various malignant tumors such as gastric cancer, liver cancer, cervical cancer, breast cancer and colorectal cancer. In this review, we compiled recent studies on the anti-tumor effects of crocin and summarized its anti-tumor mechanism for developing ideas of treating malignancies and exploring anti-tumor drugs.

## Introduction

Malignant tumours are a multifactor and multistep disease, the incidence of which has increased in recent years (Sung et al., 2021), but the pathogenesis has not been fully elucidated, so there is no effective aetiological treatment. Cancer is the second most deadly disease in the world (Fitzmaurice et al., 2017). Current cancer treatments include surgical intervention, radiation and chemotherapy drugs, which often kill healthy cells and cause a host of adverse reactions in patients. Chinese medicine plays a non-negligible role in tumor prevention and treatment with its characteristics, and it is gradually becoming an important strategy for tumor prevention and treatment because of its remarkable efficacy in improving clinical symptoms, as well as quality of life and prognosis of tumor patients.

Saffron, is native to Iran, Persia and the Mediterranean region, and was introduced to China during the Ming Dynasty (Li, 2020). Saffron is listed in the Compendium of Materia Medica as one of the best medicines, with the effect of promoting blood circulation removing blood stasis, and relieving stagnation. Crocin is the main active ingredient in saffron, which is a water-soluble carotenoid (Hashemzaei et al., 2020; Liu et al., 2021), and is widely used in medicine, food and other fields because of its unique physicochemical property. Crocin can be detected in relatively few plants, mainly including saffron in iridaceae, gardenia in rubiaceae and other plants (Guo et al., 2021). Besides, there are significant differences in the content of crocin between different plants and among different parts of the same plant. For instance, crocin is mainly found in the stigma of saffron; while it is mainly detected in the pulp of gardenia, with less content in the peel and seed of this plant (He et al., 2010).

**Figure 1:**
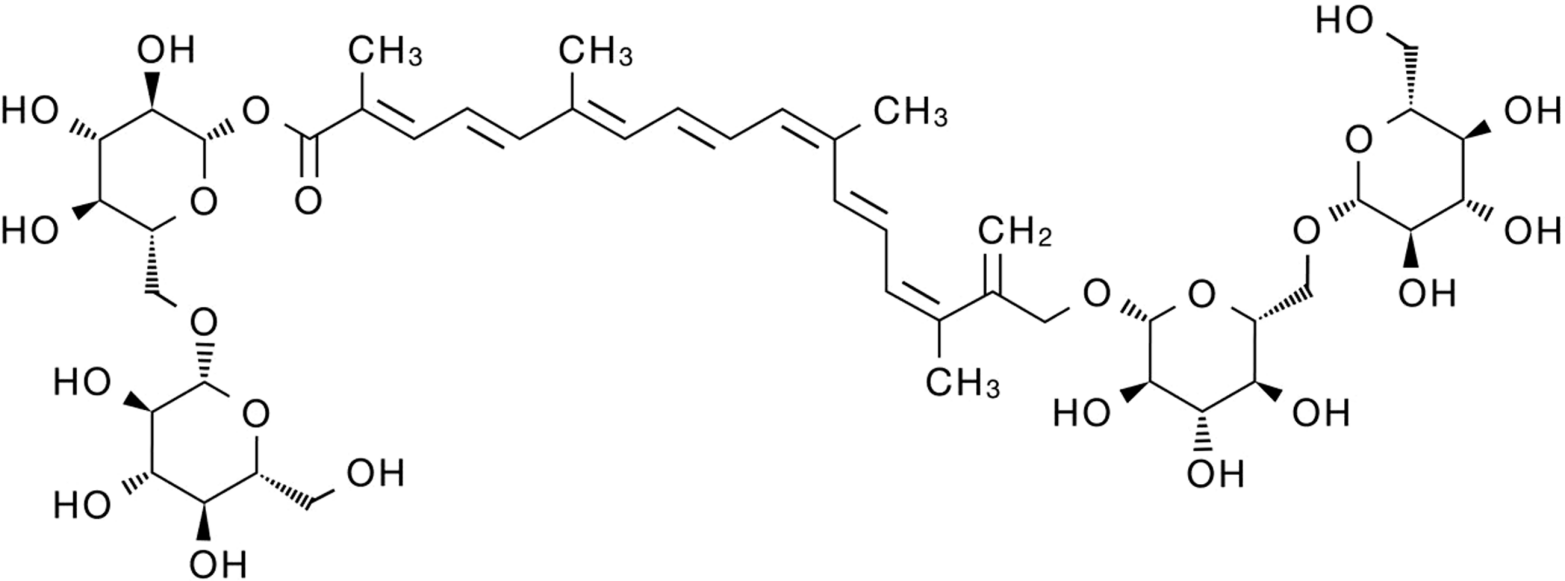
Schematic diagram of the chemical structure of crocin.

Modern pharmacological studies have shown that crocin has various pharmacological activities, including anti-oxidant (Amin et al., 2014; Finley & Gao, 2017), anti-aging (Mohammadi et al., 2018), anti-inflammatory (Li et al., 2018; Liu et al., 2018; Yarijani et al., 2017), anti-liver fibrosis (Algandaby, 2018), anti-hyperlipidemia (Sheng et al., 2006), anti-epilepsy (Zhong et al., 2022), anti-anxiety and depression (Salek et al., 2021; Xiao et al., 2020), anti-cancer (Hoshyar & Mollaei, 2017; Jiang et al., 2018; Rahaiee et al., 2017), anti-stone (Ghaeni et al., 2014) and neuro-protective (Mozaffari et al., 2019) effects. Therefore, crocin has become a hot research topic in recent years with its significant effectiveness, low toxicity and high safety. As the deep pharmacological study of crocin, it has shown significant anti-tumor biological activity and is expected to become a potential drug for the treatment of malignant tumors. Here, we review the progress of crocin’s anti-tumor mechanism in order to provide new insights for solving the problems of traditional anti-tumor drugs that have poor efficacy, high toxic and side effects and drug resistance. The chemical structure of crocin is shown below (Fig. 1).

## Induction of apoptosis in tumor cells

Apoptosis is a programmed cell death and its complexity has been the focus of numerous studies (Xu, Lai & Hua, 2019). The disruption of apoptotic mechanism is an important hallmark of cancer (Pistritto et al., 2016), and the induction of apoptosis is one of the important strategies for clinical cancer therapy. The apoptotic process can be mediated by a variety of signaling pathways.

### Tumor suppressor gene P53

P53 is a key tumor suppressor that plays a critical role in normal and cancer immunity (Agupitan et al., 2020). Loss of P53 function is a prerequisite for cancer development (Zhang et al., 2020a). p53 is highly correlated with tumors in the human body. The main biological function of p53 is to maintain the stability of cell genome, negatively regulate cell growth, and induce apoptosis. As revealed in a previous study (Jahromi et al., 2021), the growth of CO 88BV59-1 cells treated with crocin is significantly inhibited, and the expression of p53 is up-regulated. These findings indicate that crocin can promote apoptosis of CO 88BV59-1 cells in lymphoma in a time- and concentration-dependent manner by inducing the P53-dependent intrinsic pathway.

Mollaei et al. (2017) found that crocin downregulated B cell lymphoma-2 (Bcl-2) and miR-365 and up-regulated Bcl-2-associated X (Bax) and P53 in all time periods in human cervical cancer sensitive (OV2008) and resistant (C13) cell lines. The OV2008 cell line was more sensitive to crocin, which exhibited more significant change of gene expression than the C13 cell line. This study demonstrated the anti-proliferative and pro-apoptotic activity of crocin on sensitive and resistant cervical cancer cell lines, supporting that crocin could serve as an adjuvant drug to reduce chemotherapy resistance and improve therapeutic efficiency in cervical cancer treatment. In addition, MYCN gene as a transcriptional co-regulator of P53 has oncogenic effects (Agarwal et al., 2018). MYCN and p53 play opposite roles in normal development and cancer progression, but they are correlated with each other. Deng et al. (2019) observed significantly reduced expression and stability of MYCN as well as inhibited tumor cell proliferation and induced apoptosis in retinal glioma WERI-RB-1 cells after crocin treatment.

### The B lymphocytoma-2 (Bcl-2) family

BCL-2 is an important member of the BCL-2 family with anti-apoptosis effect, and its overexpression reduces apoptosis and exerts oncogenic effects. As a cell death regulator, Bax is an indispensable gateway to mitochondrial dysfunction, and it is also a major pro-apoptosis protein in the Bcl-2 family that control the apoptosis of normal cells and cancer cells (Liu et al., 2016). Bax, Bcl-2 and their ratios are considered prognostic markers of various cancers (Kulsoom et al., 2018). Evidence (Bakshi et al., 2020) has shown that crocin reduced the cell viability of pancreatic cancer BXPC3 and Capan-2 by downregulating Bcl-2 and thus activating caspases. It also modulates the expression levels of proteins including P53, P21, P27, cyclin-dependent kinase (CDK)2, c-MYC, Cytochrome C (Cyt-c)and P38 involved in cell cycle signaling. Hoshyar, Bathaie & Sadeghizadeh (2013) found that crocin has a dose- and time-dependent cytotoxic effect on gastric adenocarcinoma AGS cell lines. The increased number of G1-phase cells and activated caspases with an increased Bax/Bcl-2 ratio were observed in AGS cells after crocin treatment, which confirmed the anti-cancer effect of crocin. The apoptotic cells were significantly increased in crocin-treated AGS cells, while the above indicators (G1 phase cells, Bax/Bcl-2 ratio, and apoptotic cells) were normal in crocin-treated fibroblasts (HFSF-PI3), which indicated that crocin had no significant toxic effect on normal cells. Liu (2016) detected the evident inhibition of crocin to the proliferation of HPAC cells with a dose-dependent manner measured by MTT method. ELISA results showed that the expression of caspase-3 protein in the crocin group was significantly higher than that in the blank control group, which suggested that crocin could inhibit the proliferation of human pancreatic cancer HPAC cells and induce apoptosis of HPAC cells.

### Janus kinase/signal transducer and activator of transcription 3 (JAK/STAT3)

JAK/STAT3 signaling pathway drives tumor cell proliferation, survival, invasion, and metastasis while suppresses anti-tumor immune responses (Johnson, O’Keefe & Grandis, 2018). Therefore, JAK/STAT3 pathway inhibitors can deter tumor cell growth and stimulate anti-tumor immunity. Crocin inhibits extracellular signal-regulated kinase (ERK) and JAK/STAT signaling pathways in thyroid follicular carcinoma FTC-133, thereby inhibiting tumor cell proliferation and promoting apoptosis (Zhang et al., 2021). Wang, Ke & Shu (2020) observed that crocin reduced the phosphorylation level of STAT3 and inhibited the proliferation of colon cancer cells (HCT116) in a dose-dependent manner. In addition, it was found that crocin significantly inhibited the growth of colorectal cancer cells without toxic effects on normal cells (Aung et al., 2007).

Kim & Park (2018) found that crocin inhibited the proliferation of hepatocellular carcinoma cells (Hep3B and HepG2) and promoted apoptosis through targeting interleukin(IL)-6/STAT3 pathway by inhibiting JAK1/2 and Src kinases. In addition, crocin induced the expression of Src homology region 2 domain-containing phosphatase 1(SHP-1), leading to STAT3 dephosphorylation. While knockdown of SHP-1 expression using siRNA attenuated the effect of crocin, suggesting that SHP-1 played an important role in STAT3 signaling. Moreover, crocin downregulated the expression of cyclin D1 (proliferation), chemokine CXC motif receptor (CXCR) 4 (invasion), and vascular endothelial growth factor (VEGF)(angiogenesis) mediated by STAT3, but increased the expression of Bax (pro-apoptotic protein), indicating that crocin was associated with induction of apoptosis and inhibition of cell proliferation. This study demonstrated that crocin has anti-tumor activity through inhibiting IL-6/STAT3 signaling pathway.

### Reactive oxygen species (ROS)

**Figure 2:**
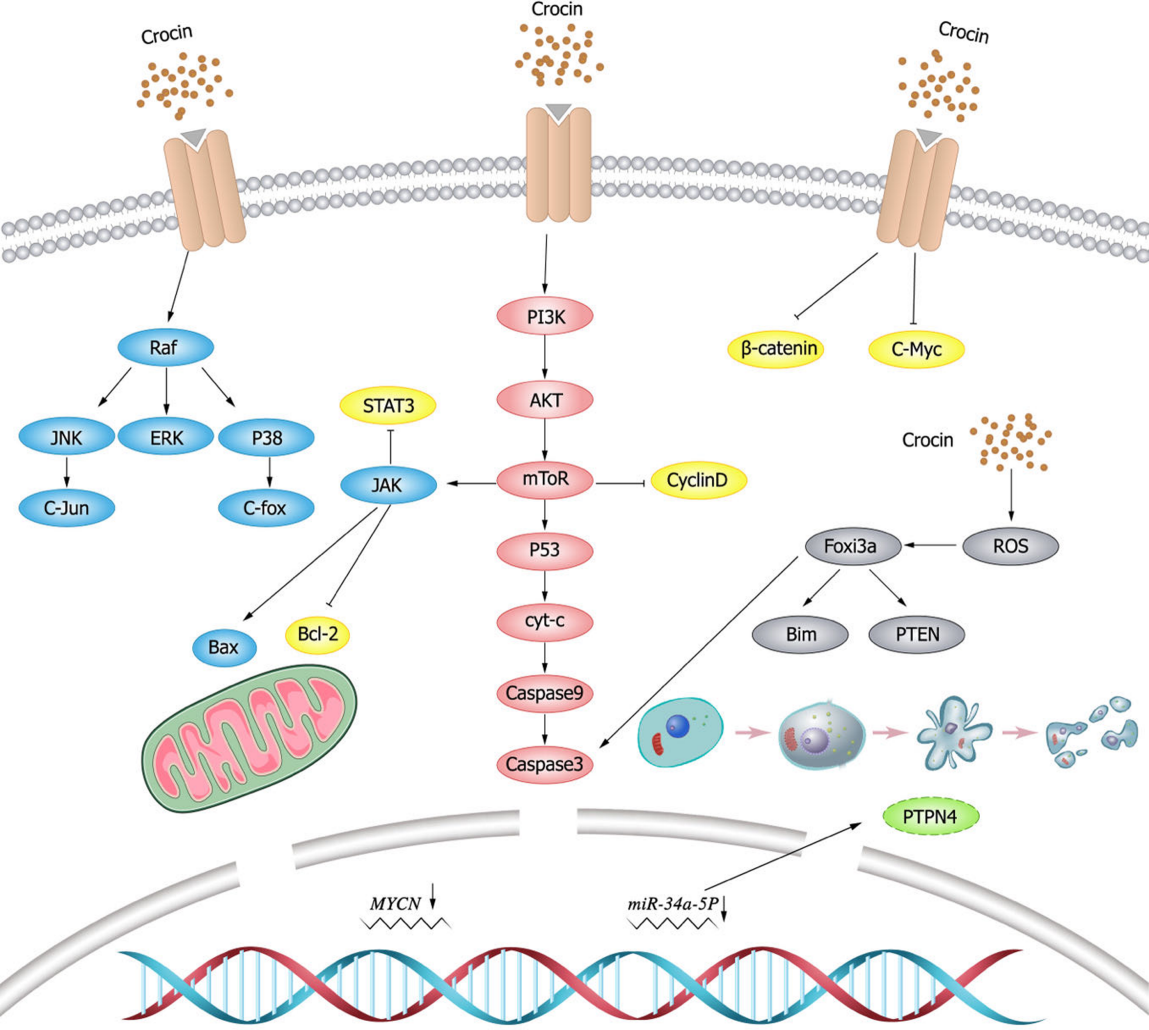
Schematic diagram of crocin inducing apoptosis of tumor cells.

ROS is a group of short-lived, highly reactive, oxygen-containing molecules that induce DNA damage and cause genotoxic stress, which plays a dual role in tumor. ROS can activate pro-tumorigenic signals, enhance tumor cell survival and proliferation, induce DNA damage and genetic instability, and initiates oxidative stress-induced tumor cell death (Moloney & Cotter, 2018; Srinivas et al., 2019). miR-34a-5p is upregulated and exerts pro-tumorigenic effects in papillary thyroid cancer (PTC), and its downregulation by crocin inhibits tumor cell growth (Tang et al., 2022). Protein tyrosine phosphatase nonreceptor (PTPN)4 is the most significantly downregulated target gene in thyroid cancer tissues, and PTPN4 can be upregulated through downregulating miR-34a-5p expression by crocin in PTC cells. Tang et al. (2022) demonstrated that crocin promoted thyroid cancer TPC-1 and IHH-4 apoptosis and increased caspase-3 activity and lactate dehydrogenase (LDH)release, which was reversed by overexpressing NAC and miR-34a, suggesting that crocin promoted ROS-mediated apoptosis in PTC cells by regulating the miR-34a-5p/PTPN4 axis. Nasimian et al. (2020) found that crocin induced ROS production, increased forkhead box O3 (FOXO3a) expression and facilitated nuclear translocation in breast cancer cell lines (MCF-7 and MDA-MB-231), which in turn increased the expression of FOXO3a target genes (Bim and phosphatase and tensin (PTEN)) and activation of caspase-3. NAC blocked the protein kinase B (AKT)/FOXO3a/Bim signaling pathway. Consequently, it could be concluded that crocin induces apoptosis in breast cancer cell lines (MCF-7 and MDA-MB-231), where ROS-activated FOXO3a cascade plays a key role in this process. Schematic diagram of crocin inducing apoptosis of tumor cells (Fig. 2).

## Inhibition of tumor cell proliferation

Cell proliferation is an important component of cell growth and differentiation (Díaz-Coránguez, Liu & Antonetti, 2019), and the abnormal cell proliferation is a typical feature of cancer (Jarrett et al., 2018), accompany with loss of differentiation and maturation for different levels. Therefore, inhibition of tumor cell proliferation is an important part of the anti-tumor treatment. At present, there are two ways to control tumor cell proliferation. One is to kill and inhibit the growth of tumor cells by improving the immunity of patients themselves, and the other is to stagnate tumor cell mitosis and control tumor growth through drug administration.

### Blocking cell cycle

Cell division is strictly regulated by multiple evolutionarily conserved cell cycle regulatory mechanisms (Matthews, Bertoli & De Bruin, 2022). Targeting cell cycle components may be an effective anti-cancer strategy. In a mouse model of breast cancer, the expression of cyclin D1 and p21 was elevated, while crocin could minish the tumor size of mouse model by inhibiting the expression of D1 and p21 (Ashrafi et al., 2015). *In vitro* experiments (Chen et al., 2019) also showed that crocin could induce cell cycle arrest in the G2/M phase of human breast cancer cells (MDA-MB-231) in a dose-dependent manner. Sun et al. (2013) showed that crocin could induce apoptosis and cell cycle arrest in the G0/G1 phase leukemia cells (HL-60) in a dose-dependent manner thereby inhibiting the proliferation of leukemia cells. Amin et al. (2016) illustrated that crocin had an anti-proliferative effect on hepatocellular carcinoma cells (HepG2) by blocking S and G2/M phases of cell cycle, inducing apoptosis and down-regulating the inflammatory response. *In vivo* experiments showed that crocin significantly inhibited the inflammatory response induced by hepatocellular carcinoma cells in rats and suggested crocin as a candidate chemopreventive agent for hepatocellular carcinoma. Xia (2015) presented that crocin markedly inhibited the growth of ovarian carcinoma cells (HO-8910) and blocked them in the G0/G1 phase. In addition, crocin also promoted apoptosis in HO-8910 cells possibly through activating the expression of p53 and Fas/APO-1, which in turn activated caspase-3 apoptotic pathway.

### Tubulin

Tubulin is one of the most important intracellular proteins, involved in almost all cell biological processes. However, tubulin was differentially expressed in normal and tumor cells, which provides ideas for the development of tumor chemotherapeutic drugs (Binarová & Tuszynski, 2019). Tubulin is also the main target of crocin in anti-tumor activity (Wang et al., 2020). Studies have shown (Zarei Jaliani et al., 2013) that crocin entering in cells can regulate cellular proteins and their functions, and can affect a variety of cellular processes by interacting with tubulin or microtubules. Hire et al. (2017) found that crocin inhibited mitosis and induced multipolar spindle formation by targeting microtubules, and thus suppressed the proliferation of breast cancer cells (HCC70 and HCC1806) and cervical cancer cells (Hela cells). Liao et al. (2022) discovered that crocin could evidently weaken viability, migration and invasion, and induce apoptosis of gastric cancer cells (MGC-803) by decreasing tubulin expression. Crocin also inhibited the proliferation of mouse gastric cancer cells (AGS) and tumor growth by decreasing tropomyosin (TPM) 4 expression, while TPM4 overexpression could resist the anti-tumor effect of crocin (Luo et al., 2021).

### Nuclear factor kappa-B (NF- *κ*B) pathway

**Figure 3:**
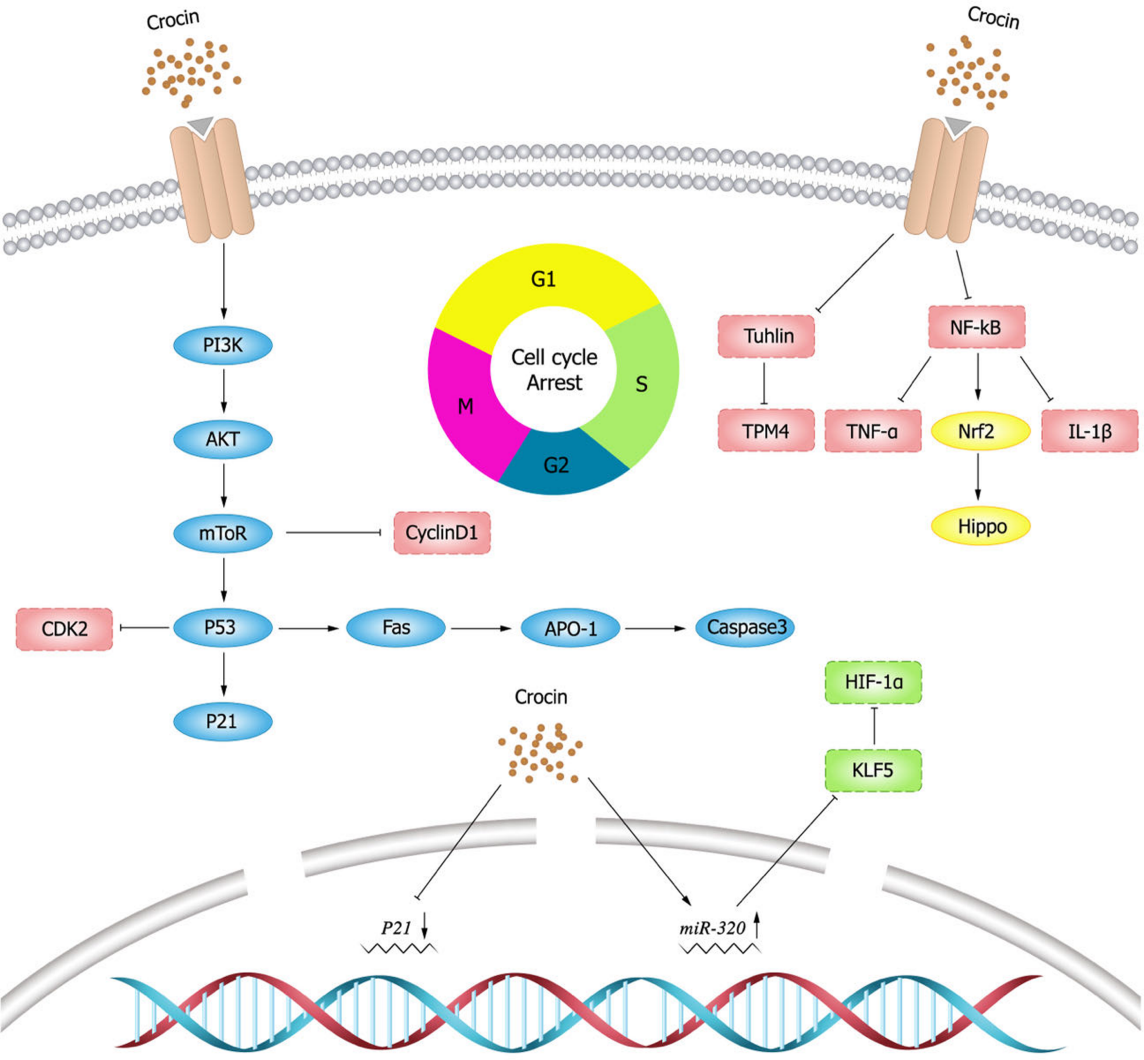
Schematic diagram of crocin inhibiting tumor cell proliferation.

NF- *κ*B has been early considered as a potential target for cancer therapy, and dysregulation of NF- *κ*B activity leads to inflammatory diseases and cancer (Yu et al., 2020). Studies have shown that crocin inhibits the activation of NF- *κ*B pathway (Hussain et al., 2021; Shi et al., 2018; Teng et al., 2021; Zhang et al., 2022b). Xu et al. (2022) showed that crocin inhibited NF- *κ*B activation and the expression of tumor necrosis factor- *α* (TNF- *α*) and IL-1 *β*, and thereby decreasing the viability and proliferation of breast cancer DA-MB-231 and MDA-MB-468 cells. Nuclear transcription factor E2 related factor 2 (Nrf2) is a major transcription factor for redox regulation and an important target of crocin. Crocin could reduce the probability of colon cancer by inhibiting NF- *κ*B expression and promoting Nrf2 expression in adenocarcinoma of colon cells (Kawabata et al., 2012). Schematic diagram of crocin inhibiting tumor cell proliferation (Fig. 3).

## Inhibition of tumor cell metastasis and invasion

Invasion and metastasis are critical characteristics of malignant tumors and play an important role in the development of cancer (Guan et al., 2020; Zeng et al., 2019). Therefore, inhibition of tumor cell invasion and metastasis plays a crucial role in cancer treatment.

### Vascular endothelial growth factor (VEGF)

Angiogenesis is essential for cancer development and growth, and VEGF is a key signal for cancer angiogenesis (Carmeliet, 2005). Evidence has shown that downregulating VEGF expression leads to tumor immunosuppression (Zhang & Brekken, 2022), and this finding is of positive value for tumor treatment. Previous studies demonstrated that crocin could downregulate VEGF expression and reduce tumor volume in mice, thus improving their survival (Farahi et al., 2021). In an experimental study by Bakshi et al. (2022), it was mentioned that crocin inhibited TNF- *α*, NF- *κ*B and VEGF pathways in colon cancer cells (HT-29 and Caco-2) in a dose-dependent manner, thereby inhibiting migration, invasion and angiogenesis of colon cancer cells. Moreover, it was found that crocin was involved in the regulation of molecules in the angiogenic pathway such as downregulating cluster of differentiation (CD)34 expression to exert anti-angiogenic effects (Chen et al., 2019). In addition, crocin could inhibit tumor cell proliferation and reduce microvessel density *in vivo*, and inhibit vascular endothelial cell proliferation, migration and tubule formation *in vitro*, revealing the main mechanism of crocin in anti-angiogenesis (Chen et al., 2016).

### Epithelial-mesenchymal transition (EMT)

EMT endows the ability of cells to invasion and metastasis, which plays a key role in cell development, as well as tissue healing, organ fibrosis and carcinogenesis. In cancer progression, EMT leads to fundamental changes in cell morphology and motility that promote cell invasion (Brabletz et al., 2021). matrix metalloproteinase (MMP) 9 is a matrix protein associated with extracellular matrix (ECM) remodeling that promotes tumor progression and regulates the activity of cell adhesion molecules and cytokines (Joseph et al., 2020). Bakshi et al. (2017b) found that crocin inhibited the expression of MMP-2, MMP-9, ERK-2, K-ras and VEGF, and attenuated the ability of melanoma cells (B16F-10) to invade, migrate, and adhere by upregulating the expression of E-cadherin. Human gastric mucosal epithelial cells (GES-1) have malignant cell characteristics such as proliferation, apoptosis, and metastatic ability. Wu & Hui (2020) found that crocin could arrest G0/G1 phase cycle, inhibit cell proliferation and promote apoptosis in GES-1 cells. In addition, crocin negatively regulated the invasive ability and EMT process of 1-methyl-3-nitroso-1-nitroguanidine (MNNG)-treated GES-1 cells, whose mechanism is possibly by inhibiting MNNG-induced malignant transformation through Nrf2/Hippo signaling pathway. Therefore, crocin may be a candidate for clinical treatment of gastric cancer. The expression levels of krüppel like factor 5 (KLF5) and hypoxia inducible factor-1 *α*(HIF-1 *α*)were elevated in gastric cancer tissues and cells, and the expression of KLF5 was positively correlated with the level of HIF-1 *α* in gastric cancer tissues. Zhou et al. (2019) demonstrated that crocin could inhibit EMT, migration and invasion of gastric cancer cells (AGS and HGC-27) by targeting miR-320/KLF5/HIF-1 *α* signaling pathway specifically *via* down-regulating the expression levels of KLF5 and HIF-1 *α* and up-regulating miR-320 expression.

### Wnt/ *β*-catenin signaling pathway

**Figure 4:**
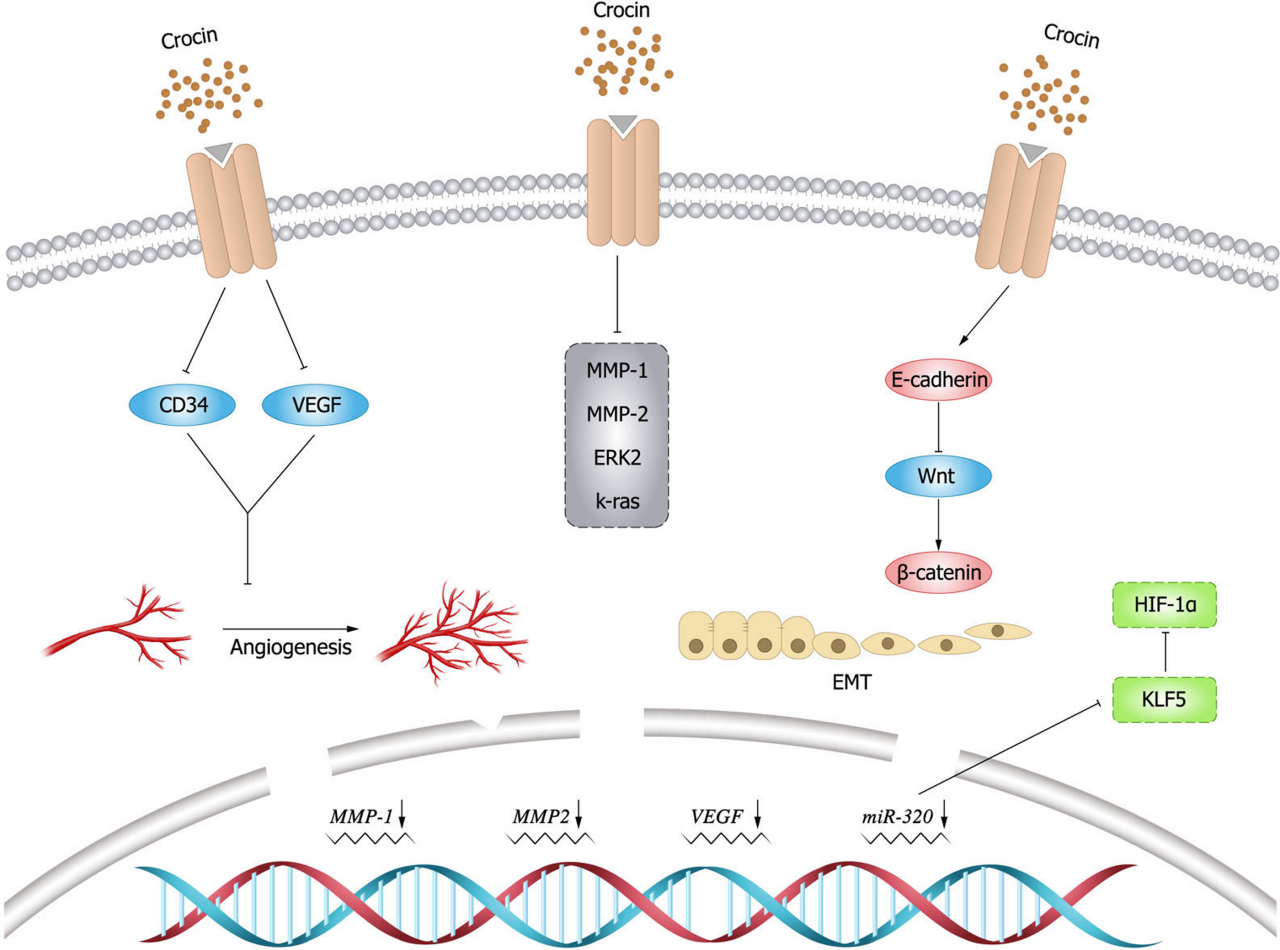
Inhibitory effect of crocin on invasion and migration of tumor cells.

*β*-catenin is particularly important for morphogenesis and cellular organization during embryogenesis (Schunk et al., 2021), and aberrant activation of *β*-catenin signal is strongly associated with increased mortality of cancer (Yu et al., 2021). Evidence showed that crocin inhibited colon cancer cell growth and invasive behavior by regulating Wnt pathway and E-calmodulin, and could significantly reduce tumor size and alleviate the symptoms alone or in combination with 5-fluorouracil (Amerizadeh et al., 2018). In breast cancer mice (BALB/c) model induced by injecting breast cancer cells (4T1), Arzi et al. (2018) found that the mice treated with crocin had increased weight, improved survival and smaller tumors. Histological examination did not reveal metastatic deposits in the liver and lungs. Compared to the untreated group, the expression of target genes within Wnt/ *β*-catenin signaling was down-regulated in tumor and lung tissues in the treated group. Moreover, crocin had no significant toxic side effects, thus crocin could be considered as a promising complementary drug against metastasis. The inhibitory effect of crocin on tumor cell invasion and migration (Fig. 4).

## Nano-liposome drug delivery enhances efficiency and reduces toxicity

The pharmacological use of carotenoid compounds is limited by their lower bioavailability and solubility. Nano-liposomal drug delivery has the advantages of good biocompatibility, broad adaptability and bioavailability. Phospholipids are cell membrane components, which are non-toxic and do not cause immune response when injected into the body. Importantly, phospholipids can protect drugs from dilution by body fluids and decomposition by enzymes in the body, which are popularly used in drug delivery (Palassi et al., 2021).

Khan et al. (2020) prepared a combination of crocin and adriamycin as nanoparticles and found that the nanoparticles were capable to produce cytotoxic effects on breast cancer cells by reducing ROS and altering mitochondrial potential, leading to apoptosis and G2/M phase cell cycle arrest. Mousavi et al. (2011) discovered that liposome encapsulation of crocin performed the enhanced cytotoxic effects on HeLa and MCF-7 cells. Rastgoo et al. (2013) developed crocin nanoliposomes for the treatment of malignant tumors and demonstrated their antitumor activity in C26 colon cancer BALB/c mice. Functionalized nanoparticles are specifically designed for drug delivery to tumor cells and can potentially enhance the anti-cancer activity of the drug. The nano-sized magnetite particles can be easily absorbed by the organisms, and when injected into animals, they degrade over time reusable by body cells. El-Kharrag et al. (2017) observed the favorable outcome of crocin-coated magnetic nanoparticles (MNPs) in treating liver cancer, shown as the regression of precancerous lesions and significant increase of apoptotic cells after treatment. The mechanism of crocin-coated MNPs may be related to the downregulation of Bcl-2, VEGF, and cyclooxygenase-2 (COX-2)expression. Crocin nanoparticles were found to have significant cytotoxic effects on breast cancer cells, suggesting that nanoparticles as drug carriers could enhance the anti-cancer effects of crocin (Hoshyar et al., 2016).

## Combination of crocin and standard drugs to increase effectiveness and reduce toxicity

Resistance of tumor cells to chemotherapeutic agents after a period of treatment is a serious problem. Commonly used chemotherapeutic drugs are prone to generate intolerance or tumor resistance, so there is an urgent need to develop new clinical drugs with increased efficacy and reduced toxicity.

Multiple resistance-associated proteins 1 and 2 (MRP1 and MRP2) are important factors responsible for the failure of cancer chemotherapy. It was shown that crocin inhibited drug resistance by downregulating the expression of MRP transporter proteins in human ovarian cancer drug-resistant cell lines (Mahdizadeh et al., 2016). In the combination therapy of crocin and cisplatin in human gastric cancer cells (BGC-823), the cancer cell proliferation was suppressed and the apoptosis was promoted (Luo et al., 2017), which may result from the increased expression of p53 and Bax and decreased expression of Bcl-2 in BGC-823 cells. Based on the results of the above experiment, it could be speculated that crocin combined with cisplatin may be a promising drug for the treatment of gastric cancer. Li et al. (2021b) found that crocin combined with cisplatin could synergistically inhibit the proliferation and growth of human cervical cancer HeLa cells, and its mechanism may be related to the regulation of apoptosis-related protein expression for promoting apoptosis and blocking cell cycle. The elevated expression levels of Cleaved Caspase-3 and Bax proteins, and the increased ratio of Bax/Bcl-2 were displayed in the combination group (crocin and cisplatin) compared with the cisplatin group. In addition, the ratio of G0/G1 phase was significantly higher while the ratio of G2/M phase was significantly lower in the combination group than that in the cisplatin group. In a study on the effect of crocin combined with cisplatin on the proliferation and apoptosis of BGC-823 cells, it was found that compared with the blank group, the combination group exhibited higher inhibition rate of cell proliferation, significantly lower expression of p-ERK1/2 and Bcl-2 proteins, and significantly higher expression of Bax and cleaved-caspase3 proteins (Xu & Luo, 2019). This suggests that crocin combined with cisplatin blocked the proliferation and promoted the apoptosis of BGC-823 cells probably by the inhibition of ERK signaling pathway.

Abdu et al. (2022) found that crocin or sorafenib or the combination of both successfully restored the normal liver structure in hepatocellular carcinoma rat model. The expression of key genes involved in carcinogenesis (TNF- *α*, p53, VEGF, and NF- *κ*B) was highly activated in hepatocellular carcinoma and was significantly attenuated after treatment with crocin or sorafenib or the combination of both. The combination group (crocin and sorafenib) showed more favorable performance in improving histopathological and inflammatory response than monotherapy (crocin or sorafenib). The administration of crocin or sorafenib alone or their combination produced cytotoxicity and anti-cancer effects on HepG2 cells, and the combination of crocin and sorafenib had synergistic antitumor effects on HepG2 cells as well. The results showed that the combination of crocin and sorafenib regimen reduced hepatotoxicity, hindered the progression of hepatocellular carcinoma, and improved liver function. The researchers proved that crocin strikingly improved the sensitivity of head and neck cancer cells (HN-5) to the combination therapy of HER1 inhibitor and radiotherapy (Torres et al., 2011). Vazifedan et al. (2017) also found that crocin inhibited the proliferation of HN-5 cells by enhancing the sensitivity of cancer cells to radiation-induced toxicity and apoptosis. Yin & Xiong (2022) showed that in cisplatin-treated cancer cells, crocin significantly reduced the levels of serum creatinine and blood urea nitrogen as well as malondialdehyde, and increased the levels of glutathione, glutathione peroxidase, catalase and superoxide dismutase in lipid peroxidation process. Crocin also significantly inhibited the activation of p38 mitogen-activated protein kinase and expression. In conclusion, crocin prevented cisplatin-induced oxidative stress and hepatorenal toxicity by attenuating the cleavage of p38 mitogen-activated protein kinase and caspase-3.

## Immunomodulatory function

Under normal circumstances, the organisms can timely recognize and remove mutated cancer cells in the body through immune surveillance. Immunosuppression is the key feature of tumor inflammatory microenvironment. Improving suppressive tumor microenvironment and reshaping tumor immunity is an effective way to improve clinical efficacy of cancer treatment (Tian et al., 2017).

Immunotherapy is of great importance for improving survival in children with acute lymphoblastic leukemia (ALL). Zhang et al. (2018) found that crocin could significantly accelerate T cell proliferation and promote IL-2 and IL-4 secretion in a dose-dependent manner. In addition, crocin could increase the CD4/CD8 ratio of T cells and reduce DNA damage in T cells without damage to T cells. Khavari et al. (2015) investigated the *in vitro* cytotoxic and apoptotic effects of saffron aqueous extract in malignant (TC-1) and non-malignant (COS-7) cell lines. The study examined the inhibitory effect of E7-NT (gp96) DNA vaccine alone and its combination with saffron extract on tumor growth with a large size. Saffron extract exerted significant anti-tumor effects by inhibiting cell growth and stimulating programmed cell death. The cytotoxicity of saffron extract on TC-1 cells was greater than that on COS-7 cells, indicating its anti-tumor activity.

An investigation showed that (Xu et al., 2012) crocin in combination with cytokines had a stronger effect to promote the proliferation of myeloid dendritic cells than crocin alone, and crocin performed a synergetic effect with cytokines to facilitate the maturation of myeloid dendritic cells. crocin-induced myeloid dendritic cells could promote the proliferation of T cells. Jiang et al. (2018) evaluated the effects of crocin on cervical cancer through examining the changes of body weight, serum enzymes, serum biochemistry, lipid peroxidation and DNA damage in male albino rats treated with crocin. The results revealed that crocin significantly reduced tumor weight, decreased the levels of alanine aminotransferase, aspartate aminotransferase, lactate dehydrogenase, alkaline phosphatase, blood urea nitrogen, creatinine, bilirubin, albumin and total protein, and increased the levels of glucose and cholesterol in rats. Hemoglobin, leukocytes, lymphocytes, neutrophils, and filler cell volume were altered after crocin treatment, as well as the occurrence of necrosis, fibrosis, mononuclear infiltration, angiogenesis, and DNA fragmentation, suggesting that crocin may be a potential anti-cancer agent for treating cervical cancer.

## Others

### Lipid metabolism pathway

A metabolic change in the lipid metabolic pathway is one of the indicators in human cancers (Merino Salvador et al., 2017). Lipid metabolism involves a complex regulatory process. Changes in lipid metabolism can not only meet the energy demand of cancer cells, but also contribute to the activation of several important carcinogenic signaling pathways (Yi et al., 2018). Cholesterol metabolites can actively promote cancer progression and suppress immune responses (Huang, Song & Xu, 2020). Triglyceride can be regarded as a biomarker for the diagnosis or prognosis of colorectal cancer (Yarla, Madka & Rao, 2022), and its metabolic dysregulation is related to the occurrence and progression of cancers. It has been revealed that crocin can effectively reduce the levels of cholesterol (Mirzaee et al., 2019; Zhang et al., 2022a) and triglyceride (Luo et al., 2019; Rahmani et al., 2019; Song et al., 2022) in serum. Hashemi, Bathaie & Mohagheghi (2020) explored the influence of crocin on the lipid content in breast cancer. It was found that crocin can inhibit tumor progression by reducing cholesterol and triglyceride in the serum of tumor-bearing mice and MDA-MB-231 and MCF-7 cell lines in breast cancer. In summary, the lipid-lowering effect of crocin is of great significance for the prevention of cancer progression, metastasis, and invasion.

### Autophagy

Autophagy is an intracellular degradation process (Li et al., 2017), and it is crucial for maintaining cellular homeostasis (Li et al., 2021a). Autophagy is closely related to malignant transformation, tumor progression, and treatment (Yamazaki et al., 2021). The dysregulation of miR-320a has been confirmed in various malignant tumors (Yang et al., 2014). In cutaneous squamous cell carcinoma (cSCC), the expression of miR-320a is significantly up-regulated, while that of ATG2B is significantly down-regulated. miR-320a can inhibit autophagy, while crocin can significantly inhibit the growth of cSCC cells and induce apoptosis through autophagy. Therefore, the inhibitory effect of crocin on cSCC may be obtained by targeting the ATG2B/miR-320a pathway (Bi et al., 2021). Crocin can significantly promote autophagy, accompanied by the activation of adenosine 5′-monophosphatate (AMP)-activated protein kinase (AMPK). Based on that, Zeng et al. (2016) maintained that the autophagy and apoptosis of cells induced by crocin correlated with AMPK, AKT, and mTOR. Zhang et al. (2020b) found that the proportion of LC3II/I, Beclin1, and ATG7 in SiHa cells in cervical cancer increased significantly after being treated with crocin. Hence, crocin may promote autophagy and apoptosis by reducing AMPK and mTOR signal transduction. LC3-II is expressed during autophagy, and its accumulation is an indicator of autophagy (Amelio, Melino & Knight, 2011; Klionsky, Cuervo & Seglen, 2007). Amin et al. (2015) found that the proliferation of HCT116 cells was inhibited after being treated with crocin in colorectal cancer. Further, the expression of LC3-II protein was significantly up-regulated in crocin-treated cells. These results suggested that the inhibition of crocin on HCT116 cells might be realized by promoting the expression of LC3-II and triggering autophagy.

However, autophagy may play different roles at different stages of cancer development. As per another study (Feidantsis et al., 2018), crocin can enhance the heat shock response of myocardial cells and inhibit apoptosis by targeting autophagy. In short, autophagy plays a role in tumor inhibition and promotion. Therefore, it is necessary to further clarify the exact mechanism of autophagy in cancers.

### Chemoprevention

Natural products have always been considered one of the most important resources of anticancer agents. Chemoprevention refers to the prevention or inhibition of canceration through the intervention of natural or synthetic products. According to the theory of traditional Chinese medicine (TCM), the occurrence of tumors is related to qi-stagnancy and blood stasis in the human body. Since saffron has an effect on activating blood circulation to dissipate blood stasis, this herbal medicine can be applied to the prevention and treatment of malignant tumors. As a carotenoid, crocin is an active component in saffron. According to modern medicine, carotenoids are common antioxidants and have anti-mutagenic and immunomodulatory functions (Brewczyński et al., 2021). It has also been demonstrated in an epidemiological study (Milani et al., 2017) that there is a correlation between carotenoid intake in diet and cancer risks.

**Figure 5:**
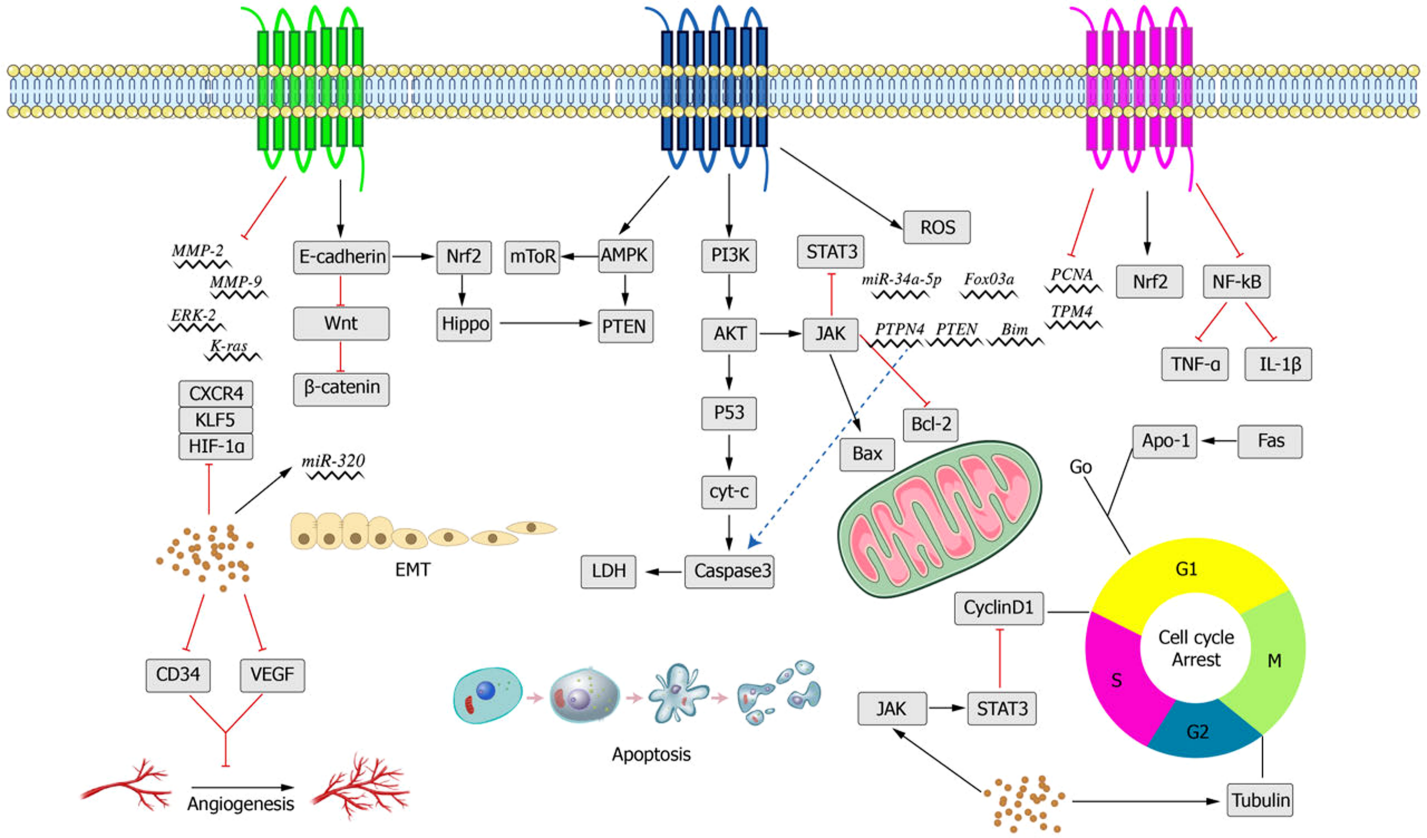
Main anti-cancer molecular pathways mediated by crocin.

In addition, crocin can significantly inhibit the expression of JNK in the liver, and up-regulate the expression of apoptosis-inducing ligand, caspase-8 protein, and p53 gene (Elsherbiny et al., 2020). Therefore, it is considered that crocin can reduce the probability of liver cancer induced by experiments *via* regulating oxidation/apoptosis signals. Sajjadi & Bathaie (2017) found that crocin could exert protective effects on rats in the occurrence of breast cancer induced by N-Nitroso-N-methylurea (NMU). The incidence of breast cancer in rats was 77% after NMU injection, while that decreased to 45% after crocin treatment. The extract of saffron also inhibited the formation of papilloma induced by 7,12-dimethylbenz [a] anthracene and reduced the average number of papilloma in mice (Randhawa & Alghamdi, 2011). Moreover, Bakshi et al. (2017a) and Bakshi et al. (2017b) found in a mouse model of melanoma that crocin treatment can significantly reduce the tumor burden, lengthen the tumor dormancy, reduce the tumor growth rate, and prolong the average survival of mice. These results corroborate that crocin in saffron has obvious chemoprevention effects on tumors.

saffron has been applied in the treatment of various diseases, including cancer, for a long time (Li, Lee & Wu, 2004). The findings of some experimental studies *in vivo* and *in vitro* mentioned above indicate that crocin, as a natural active ingredient, has fewer toxic and side effects, strong efficacy, and multiple targets in relevant treatment.A summary of the anti-tumor effects of crocin and its anti-tumor mechanism are shown below (Tables 1 and 2 and Fig. 5).

**Table 1:** Antitumor effect and signaling pathway of crocin *in vitro*.

**Work way**	**Cell type**	**Concentrations**	**Signaling pathways**	**Selective toxicity**	**REF**
Apoptosis	CO 88BV59-1	80 µM	p53	Yse (B-lymphocyte)	28
	OV2008	1.5mg/ml	Bax/Bcl-2, p53	Not mentioned in the article	29
	WERI-RB-1	20 µM	MYCN	Not mentioned in the article	31
	BXPC3, Capan-2	10, 20, 40 µg/mL	Bcl-2, caspase	Not mentioned in the article	34
	AGS	2.5 mg/mL	Caspases, Bax/Bcl-2	Yes (HFSF-PI3)	35
	HPAC	2.5, 5, 10mg/ml	Caspases-3	Not mentioned in the article	36
	FTC-133	15, 20 µM	ERK, JAK/STAT	Yes (PCCL3)	38
	HCT116	135.6, 271.18 µM	STAT3	Not mentioned in the article	39
	HepG2	20 µM	IL-6/STAT3	Not mentioned in the article	41
	Hep3B	20 µM	STAT3, Bax	Not mentioned in the article	41
	TPC-1 and IHH-4	40 µM	caspase-3, LDH	Not mentioned in the article	44
	MCF-7, MDA-MB-231	2.7, 3 mM	ROS, FOXO3a	Not mentioned in the article	45
Proliferation	MDA-MB-231	5.97 mg/mL	CD34	No (HUVEC)	50
	HL-60	5.0 mg/mL		Not mentioned in the article	51
	HepG2	1mM	IL-8	Not mentioned in the article	52
	HO-8910	1.0 mmol/L		Not mentioned in the article	53
	HCC70, HCC1806, HeLa	50 µM	tubulin	Yes (CCD1059sk)	57
	MGC-803	100 ng/m L	*α*-Tubulin	Not mentioned in the article	58
	AGS	2, 4, 6 mg/mL	TPM4	Not mentioned in the article	59
	DA-MB-231, MDA-MB-468	4 mg/mL	NF- *κ*B, TNF- *α*, IL-1 *β*	Not mentioned in the article	65
	GES-1	3, 10 µM	Nrf2/Hippo	Not mentioned in the article	77
Metastasis	HT-29, Caco-2	10, 20, 40 µg/mL	VEGF	Yes (HCEC)	72
	MDA-MB-231	5.97 mg/mL	CD34	No (HUVEC)	50
	GES-1	10 µM	Nrf2/Hippo	Not mentioned in the article	77
	AGS, HGC-27	2, 3 µM	miR-320/KLF5/HIF-1 *α*	Yes (GES-1)	78
Nanoliposomes administration mode	MDA-MB-231, MCF-7	1, 5, 25 µM	ROS	Yes (HBL-100, H9c2)	84
	HeLa, MCF-7	1.2 mM	LC3II/I, Beclin1 ATG7	Yes (L929)	85
	HepG2	3 mg/ml	Bcl-2, VEGF, COX-2	Not mentioned in the article	87
	MCF-7	3 mg/ml		Not mentioned in the article	88
Combination with cisplatin	BGC-823	8, 16 mg/mL	Bax/Bcl-2, p53	Not mentioned in the article	90
	HeLa	400 µg/mL	Cleaved Caspase-3, Bax/Bcl-2	Not mentioned in the article	91
	BGC-823	8 mg/ml	p-ERK1/2, Bcl-2, Bax, cleaved-caspase3	Not mentioned in the article	92
Combination with sorafenib	HepG2	50-300 µM	TNF- *α*, p53, VEGF NF- *κ*B	Not mentioned in the article	93
Enhanced radiation sensitivity	HN-5	12.5-1000 µg/mL		Not mentioned in the article	95
Metabolism of fat	MDA-MB-231, MCF-7	2-5 mg/ml	Chl/TG	Not mentioned in the article	110
Autophagy	A431, SCL-1	1, 2, 4 mM	ATG2B/miR-320a	Not mentioned in the article	115
	SiHa	2, 4, 8, 16 mM	AMPK mTOR	Not mentioned in the article	117
	HCT116	10mM	LC3-II	Not mentioned in the article	120

**Table 2:** Antitumor effect and signaling pathway of crocin *in vivo*.

**Work way**	**Tissue type**	**Dosage**	**Signaling pathways**	**Selective toxicity**	**REF**
Proliferation	Breast tumors	200 mg/kg/d	CyclinD1, P21	Not mentioned in the article	49
	Colonic adenocarcinomas	50, 100, 200 ppm	NF- *κ*B, Nrf2	Yes	66
Metastasis	Melanoma	250, 500 µg/kg/d	MMP-2, MMP-9, VEGF	Not mentioned in the article	76
	Breast cancer	200 mg/kg/d	Wnt/ *β*-catenin	Not mentioned in the article	82
Nanoliposomes administration mode	Colon carcinoma	50, 100 mg/kg/d		Not mentioned in the article	86
Combination with cisplatin	Kunming mice	6.25, 12.5 mg/kg/d	P38, p53, caspase-3	Not mentioned in the article	96
Autophagy	Cardiac tissues	50 mg/kg/d	AMPK, AKT, mTOR	Not mentioned in the article	116

## Safety and Tolerance Clinical Trials

Safety and tolerance of drugs are major concerns in relevant clinical trials. In a clinical trial on the subjects with mild to moderate sleep disorders related to anxiety (Pachikian et al., 2021), the sleep quality in the treatment group (crocin was administered at 15.5 mg daily for 6 weeks) was improved, while that in the placebo group did not change significantly, and no serious adverse events were reported. In another trial on diabetic macular edema (Sepahi et al., 2018), crocin was administered as a supplement at 15mg daily for 3 consecutive months. It was observed that crocin (15mg) contributed to reducing inflammatory responses in patients with diabetic maculopathy. It is demonstrated that crocin can be used as an effective antioxidant and neuroprotective agent with favorable tolerance. Chemotherapy-induced peripheral neuropathy (CIPN) is one of the complications in patients treated with anticancer drugs. In an 8-week clinical trial (Bozorgi et al., 2021), crocin achieved excellent efficacy in relieving CIPN symptoms of cancer patients treated with chemotherapy drugs, mainly manifested in analgesic effects. This suggests that crocin can be used as a supplement to anti-tumor drugs.

In a clinical study investigating the effect of saffron on plasma cholesterol ester transfer protein (CETP) levels in patients with metabolic syndrome, participants were randomly assigned to either a saffron group (30 mg/d) or a placebo group (Javandoost et al., 2017). The study lasted for eight weeks during which lipid metabolism markers and CETP levels were recorded. Results showed that, compared to the control group, the saffron group demonstrated improved CETP levels, triglyceride, and low-density lipoprotein cholesterol. This improvement trend strengthened with the extended treatment time. Participants were monitored for safety during the study period, with no serious adverse reactions or side effects identified. Therefore, this study suggests that saffron is relatively safe at doses used.

Although this trial provided helpful information, it was limited to patients with metabolic syndrome; thus, further studies are necessary to explore its applicability to healthy individuals. Additionally, the study did not consider the effects of different doses and durations on treatment efficacy. Therefore, additional laboratory tests, animal experiments, and clinical trials are required to more accurately evaluate the effectiveness and side effects of saffron as a metabolic syndrome treatment.

A randomized, double-blind, placebo-controlled trial was conducted to evaluate the effects of saffron on inflammation markers, lipid levels, insulin, and cardiac protection in women diagnosed with polycystic ovary syndrome (PCOS) (Rahimi, Shams & Aslani, 2022). In this study, 60 participants with PCOS were randomly assigned to receive either 15 milligrams of saffron per day or a placebo. The trial lasted for 12 weeks, during which information regarding participants’ biochemical markers and heart health was recorded. Results demonstrated reductions in low-density lipoprotein cholesterol, triglycerides, and inflammation markers in the saffron group compared to the placebo group. Additionally, the saffron group showed an increase in high-density lipoprotein, cholesterol and heart protection. No adverse reactions or side effects were observed in the saffron group. Overall, the study suggests that saffron improves blood lipid levels, inflammation, and heart health in women with PCOS. However, due to the limited sample size, additional research is needed to explore other potential factors, such as different doses and durations of use, to determine the optimal use of saffron in PCOS treatment.

In a double-blind, randomized controlled trial (Kazemi et al., 2021), researchers compared saffron with fluoxetine, a commonly used antidepressant and anti-obsessive-compulsive medication, in the treatment of mild to moderate obsessive-compulsive disorder (OCD). This study randomly assigned 60 patients with mild to moderate OCD to receive either a 15 mg daily saffron supplement or a 20 mg daily fluoxetine dosage. The trial lasted for eight weeks, during which information about participants’ obsessive symptoms and mental state was recorded. Both the saffron and fluoxetine groups showed significant clinical improvement, but there was no significant difference between the two groups. However, it is important to note that fewer adverse reactions occurred in the saffron group than in the fluoxetine group, suggesting that saffron may have a higher safety profile compared to fluoxetine. Consequently, this study suggests that saffron may be an alternative treatment for OCD and a better option for patients who are intolerant to or experience adverse reactions to fluoxetine. Although these results are encouraging, the limited sample size emphasizes the need for further studies on other potential factors, such as different doses and duration of use, to determine the optimal use of saffron in the treatment of OCD.

## Conclusions and Outlooks

It is well-known that it is difficult to completely resolve malignant tumors. Therefore, it is required to conduct scheduled, reasonable, and standardized treatment according to the biological characteristics of tumors, patients’ conditions, disease stages, and development trends. For the treatment of malignant tumors, it is necessary to take account of the effectiveness and safety of treatment and the survival time of patients. Furthermore, the treatment costs, subjective feelings, and quality of life of patients shall also be considered during treatment.

Crocin, as a type carotenoid with a great medicinal value, has wide anti-tumor efficacy and inhibitory effects in a variety of malignant tumors, such as gastric cancer, liver cancer, cervical, breast cancer and colorectal cancer. Crocin exerts its anti-tumor effect mainly by promoting apoptosis, inhibiting malignant proliferation, and interfering cell cycle of tumor cells.

Although crocin is well tolerated and has high safety (Javandoost et al., 2017; Kazemi et al., 2021; Rahimi, Shams & Aslani, 2022), the research on crocin only focuses on *in vitro* experiments and rodent models, and further studies are needed to determine the optimal dose and verify its safety for human use. Favorable anti-tumor effects have been observed in various tumors, but the mechanism has not been clearly uncovered. The bioavailability of carotenoid components is not high due to complex factors affecting their absorption, decomposition, transport and storage. Crocin has low stability and poor absorption, which affects its bioavailability (Puglia et al., 2019). Research has shown that it is not well-absorbed after oral administration and quickly hydrolyzes to crocetin in the intestine (Xi et al., 2007). However, under *in vitro* simulated gastrointestinal digestion conditions, the bioavailability of crocin can reach up to 50% (Kyriakoudi et al., 2013), suggesting that its bioavailability can be improved under certain conditions. Nanocarriers can overcome the restriction of drug action by body barriers (such as the blood–brain barrier and blood–eye barrier) and can reduce drug doses, improve drug availability and reduce side effects after modification by targeted groups. Moreover, the extraction rate of crocin is low and the cost is high. Therefore, it is necessary to develop an efficient extraction process to further expand its application in the fields of clinical medicine, food and drug.

As a natural compound, saffron has shown promising anticancer effects; however, its underlying mechanism and efficacy require further exploration. Future research directions may include the following aspects:

Mechanism of action: Investigation into the specific mechanisms of saffron in tumour therapy by elucidating its regulatory role in apoptosis, proliferation, invasion, metastasis, glucolipid metabolism, and other cellular processes.

Rational drug use: Investigation into the safety, toxicity, and side effects of saffron, as well as its indications for different types of cancer, to develop rational drug-use plans that improve clinical outcomes.

Development of novel saffron derivatives: Use of molecular modification, synthetic biology, and other technologies to design and synthesize novel saffron derivatives with enhanced anticancer activity and pharmacological properties while reducing adverse reactions.

Drug combination: Study the combined application of saffron with other drugs to explore its synergistic effect, advantages, and expand its application range in tumour therapy.

Basic research: Conduct in-depth exploration of the interactions between saffron and tumor cells, immune system, and other related biological systems at a basic research level to improve our knowledge and understanding of the anticancer mechanisms of saffron.

Given the increasing demand for effective tumor therapies and the constant progress in science and technology, the research and development of saffron in the field of anticancer will remain a hot spot and an essential direction for future study. In summary, crocin has a great potential in defeating tumors. Especially, the processed and modified nanoliposome delivery for crocin is expected to become a novel anti-tumor drug. It is believed that the anti-tumor pharmacological mechanism of crocin will be further explored and verified in the future.

## Abbreviations

Bcl-2: B cell lymphoma-2
Bax: Bcl-2-associated X
CDK: Cyclin-dependent kinase
Cyt-c: Cytochrome C
JAK: Janus kinase
STAT3: Signal transducer and activator of transcription 3
ERK: extracellular signal-regulated kinase
SHP-1: Src homology region 2 domain-containing phosphatase 1
CXCR: Chemokine CXC motif receptor
VEGF: Vascular endothelial growth factor
IL: Interleukin
ROS: Reactive oxygen species
PTC: Papillary thyroid cancer
PTPN: Protein tyrosine phosphatase nonreceptor
LDH: Lactate dehydrogenase
FOXO3a: Forkhead box O3
PTEN: Phosphatase and tensin
AKT: Protein kinase B
TPM: Tropomyosin
NF- *κ*B: Nuclear factor kappa-B
Nrf2: Nuclear transcription factor E2 related factor 2
EMT: Epithelial-mesenchymal transition
MMP: Matrix metalloproteinase
MNNG: 1-methyl-3-nitroso-1-nitroguanidine
KLF5: Krüppel like factor 5
HIF-1 *α*: Hypoxia inducible factor-1 *α*
MNPs: Magnetic nanoparticles
COX-2: Cyclooxygenase-2
MRP: Multiple resistance-associated proteins
TNF- *α*: Tumor necrosis factor- *α*
CD: Cluster of differentiation
AMPK: Adenosine 5′-monophosphate (AMP)-activated protein kinase
mTOR: Mammalian target of rapamycin
NMU: N-methyl-N-nitrosourea
OCD: Obsessive-compulsive disorder
PCOS: Polycystic ovary syndrome
CETP: Cholesterol ester transfer protein
CIPN: Chemotherapy-induced peripheral neuropathy

## Additional Information and Declarations

### Data Availability

The following information was supplied regarding data availability: This is a literature review and did not use raw data.

## References

[ref-1] Abdu S, Juaid N, Amin A, Moulay M, Miled N (2022). Therapeutic effects of crocin alone or in combination with sorafenib against hepatocellular carcinoma: *in vivo* & *in vitro* insights. Antioxidants.

[ref-2] Agarwal S, Milazzo G, Rajapakshe K, Bernardi R, Chen Z, Barbieri E, Koster J, Perini G, Coarfa C, Shohet JM (2018). MYCN acts as a direct co-regulator of p53 in MYCN amplified neuroblastoma. Oncotarget.

[ref-3] Agupitan AD, Neeson P, Williams S, Howitt J, Haupt S, Haupt Y (2020). P53: a guardian of immunity becomes its saboteur through mutation. International Journal of Molecular Sciences.

[ref-4] Algandaby MM (2018). Antifibrotic effects of crocin on thioacetamide-induced liver fibrosis in mice. Saudi Journal of Biological Sciences.

[ref-5] Amelio I, Melino G, Knight RA (2011). Cell death pathology: cross-talk with autophagy and its clinical implications. Biochemical and Biophysical Research Communications.

[ref-6] Amerizadeh F, Rezaei N, Rahmani F, Hassanian SM, Moradi-Marjaneh R, Fiuji H, Boroumand N, Nosrati-Tirkani A, Ghayour-Mobarhan M, Ferns GA, Khazaei M, Avan A (2018). Crocin synergistically enhances the antiproliferative activity of 5-flurouracil through Wnt/PI3K pathway in a mouse model of colitis-associated colorectal cancer. Journal of Cellular Biochemistry.

[ref-7] Amin B, Abnous K, Motamedshariaty V, Hosseinzadeh H (2014). Attenuation of oxidative stress, inflammation and apoptosis by ethanolic and aqueous extracts of *Crocus sativus* L. stigma after chronic constriction injury of rats. Anais da Academia Brasileira de Ciencias.

[ref-8] Amin A, Bajbouj K, Koch A, Gandesiri M, Schneider-Stock R (2015). Defective autophagosome formation in p53-null colorectal cancer reinforces crocin-induced apoptosis. International Journal of Molecular Sciences.

[ref-9] Amin A, Hamza AA, Daoud S, Khazanehdari K, Hrout AA, Baig B, Chaiboonchoe A, Adrian TE, Zaki N, Salehi-Ashtiani K (2016). Saffron-based crocin prevents early lesions of liver cancer: *in vivo, in vitro* and network analyses. Recent Patents on Anti-Cancer Drug Discovery.

[ref-10] Arzi L, Farahi A, Jafarzadeh N, Riazi G, Sadeghizadeh M, Hoshyar R (2018). Inhibitory effect of crocin on metastasis of triple-negative breast cancer by interfering with Wnt/ *β*-catenin pathway in murine model. DNA and Cell Biology.

[ref-11] Ashrafi M, Bathaie SZ, Abroun S, Azizian M (2015). Effect of crocin on cell cycle regulators in N-Nitroso-N-methylurea-induced breast cancer in rats. DNA and Cell Biology.

[ref-12] Aung HH, Wang CZ, Ni M, Fishbein A, Mehendale SR, Xie JT, Shoyama CY, Yuan CS (2007). Crocin from *Crocus sativus* possesses significant anti-proliferation effects on human colorectal cancer cells. Experimental Oncology.

[ref-13] Bakshi HA, Hakkim FL, Sam S, Javid F (2017a). Role of dietary crocin in *in vivo* melanoma tumor remission. Asian Pacific Journal of Cancer Prevention.

[ref-14] Bakshi HA, Hakkim FL, Sam S, Javid F, Rashan L (2017b). Dietary crocin reverses melanoma metastasis. The Journal of Biomedical Research.

[ref-15] Bakshi HA, Quinn GA, Nasef MM, Mishra V, Aljabali AAA, El-Tanani M, Á Serrano-Aroca, Webba Da Silva M, McCarron PA, Tambuwala MM (2022). Crocin inhibits angiogenesis and metastasis in colon cancer *via* TNF- *α*/NF-kB/VEGF pathways. Cells.

[ref-16] Bakshi HA, Zoubi MSA, Hakkim FL, Aljabali AAA, Rabi FA, Hafiz AA, Al-Batanyeh KM, Al-Trad B, Ansari P, Nasef MM, Charbe NB, Satija S, Mehta M, Mishra V, Gupta G, Abobaker S, Negi P, Azzouz IM, Dardouri AAK, Dureja H, Prasher P, Chellappan DK, Dua K, Webba da Silva M, El Tanani M, McCarron PA, Tambuwala MM (2020). Dietary crocin is protective in pancreatic cancer while reducing radiation-induced hepatic oxidative damage. Nutrients.

[ref-17] Bi X, Jiang Z, Luan Z, Qiu D (2021). Crocin exerts anti-proliferative and apoptotic effects on cutaneous squamous cell carcinoma *via* miR-320a/ATG2B. Bioengineered.

[ref-18] Binarová P, Tuszynski J (2019). Tubulin: structure, functions and roles in disease. Cells.

[ref-19] Bozorgi H, Ghahremanfard F, Motaghi E, Zamaemifard M, Zamani M, Izadi A (2021). Effectiveness of crocin of saffron (*Crocus sativus* L.) against chemotherapy-induced peripheral neuropathy: a randomized, double-blind, placebo-controlled clinical trial. Journal of Ethnopharmacology.

[ref-20] Brabletz S, Schuhwerk H, Brabletz T, Stemmler MP (2021). Dynamic EMT: a multi-tool for tumor progression. The EMBO Journal.

[ref-21] Brewczyński A, Jabłońska B, Kentnowski M, Mrowiec S, Składowski K, Rutkowski T (2021). The association between carotenoids and head and neck cancer risk. Nutrients.

[ref-22] Carmeliet P (2005). VEGF as a key mediator of angiogenesis in cancer. Oncology.

[ref-23] Chen SS, Gu Y, Lu F, Qian DP, Dong TT, Ding ZH, Zhao S, Yu ZH (2019). Antiangiogenic effect of crocin on breast cancer cell MDA-MB-231. Journal of Thoracic Disease.

[ref-24] Chen SS, Zhao S, Gu Y, Jiang EZ, Zhu JU, Yu ZH (2016). Experimental study on antiangiogenic effect of crocin on breast cancer. Chinese Clinical Oncology.

[ref-25] Deng L, Li J, Lu S, Su Y (2019). Crocin inhibits proliferation and induces apoptosis through suppressing MYCN expression in retinoblastoma. Journal of Biochemical and Molecular Toxicology.

[ref-26] Díaz-Coránguez M, Liu X, Antonetti DA (2019). Tight junctions in cell proliferation. International Journal of Molecular Sciences.

[ref-27] El-Kharrag R, Amin A, Hisaindee S, Greish Y, Karam SM (2017). Development of a therapeutic model of precancerous liver using crocin-coated magnetite nanoparticles. International Journal of Oncology.

[ref-28] Elsherbiny NM, Eisa NH, El-Sherbiny M, Said E (2020). Chemo-preventive effect of crocin against experimentally-induced hepatocarcinogenesis *via* regulation of apoptotic and Nrf2 signaling pathways. Environmental Toxicology and Pharmacology.

[ref-29] Farahi A, Abedini MR, Javdani H, Arzi L, Chamani E, Farhoudi R, Talebloo N, Hoshyar R (2021). Crocin and Metformin suppress metastatic breast cancer progression *via* VEGF and MMP9 downregulations: *in vitro* and *in vivo* studies. Molecular and Cellular Biochemistry.

[ref-30] Feidantsis K, Mellidis K, Galatou E, Sinakos Z, Lazou A (2018). Treatment with crocin improves cardiac dysfunction by normalizing autophagy and inhibiting apoptosis in STZ-induced diabetic cardiomyopathy. Nutrition, Metabolism and Cardiovascular Diseases.

[ref-31] Finley JW, Gao S (2017). A Perspective on *Crocus sativus* L. (Saffron) constituent crocin: a potent water-soluble antioxidant and potential therapy for alzheimer’s disease. Journal of Agricultural and Food Chemistry.

[ref-32] Fitzmaurice C, Allen C, Barber RM, Barregard L, Bhutta ZA, Brenner H, Dicker DJ, Chimed-Orchir O, Dandona R, Dandona L, Fleming T, Forouzanfar MH, Hancock J, Hay RJ, Hunter-Merrill R, Huynh C, Hosgood HD, Johnson CO, Jonas JB, Khubchandani J, Kumar GA, Kutz M, Lan Q, Larson HJ, Liang X, Lim SS, Lopez AD, MacIntyre MF, Marczak L, Marquez N, Mokdad AH, Pinho C, Pourmalek F, Salomon JA, Sanabria JR, Sandar L, Sartorius B, Schwartz SM, Shackelford KA, Shibuya K, Stanaway J, Steiner C, Sun J, Takahashi K, Vollset SE, Vos T, Wagner JA, Wang H, Westerman R, Zeeb H, Zoeckler L, Abd-Allah F, Ahmed MB, Alabed S, Alam NK, Aldhahri SF, Alem G, Alemayohu MA, Ali R, Al-Raddadi R, Amare A, Amoako Y, Artaman A, Asayesh H, Atnafu N, Awasthi A, Saleem HB, Barac A, Bedi N, Bensenor I, Berhane A, Bernabé E, Betsu B, Binagwaho A, Boneya D, Campos-Nonato I, Castañeda Orjuela C, Catalá-López F, Chiang P, Chibueze C, Chitheer A, Choi JY, Cowie B, Damtew S, Neves Jdas, Dey S, Dharmaratne S, Dhillon P, Ding E, Driscoll T, Ekwueme D, Endries AY, Farvid M, Farzadfar F, Fernandes J, Fischer F, GH TT, Gebru A, Gopalani S, Hailu A, Horino M, Horita N, Husseini A, Huybrechts I, Inoue M, Islami F, Jakovljevic M, James S, Javanbakht M, Jee SH, Kasaeian A, Kedir MS, Khader YS, Khang YH, Kim D, Leigh J, Linn S, Lunevicius R, Razek HMAEl, Malekzadeh R, Malta DC, Marcenes W, Markos D, Melaku YA, Meles KG, Mendoza W, Mengiste DT, Meretoja TJ, Miller TR, Mohammad KA, Mohammadi A, Mohammed S, Moradi-Lakeh M, Nagel G, Nand D, Nguyen QLe, Nolte S, Ogbo FA, Oladimeji KE, Oren E, Pa M, Park EK, Pereira DM, Plass D, Qorbani M, Radfar A, Rafay A, Rahman M, Rana SM, Søreide K, Satpathy M, Sawhney M, Sepanlou SG, Shaikh MA, She J, Shiue I, Shore HR, Shrime MG, So S, Soneji S, Stathopoulou V, Stroumpoulis K, Sufiyan MB, Sykes BL, Tabarés-Seisdedos R, Tadese F, Tedla BA, Tessema GA, Thakur JS, Tran BX, Ukwaja KN, Uzochukwu BSC, Vlassov VV, Weiderpass E, Terefe MWubshet, Yebyo HG, Yimam HH, Yonemoto N, Younis MZ, Yu C, Zaidi Z, Zaki MES, Zenebe ZM, Murray CJL, Naghavi M (2017). Global. regional, and national cancer incidence, mortality, years of life lost, years lived with disability, and disability-adjusted life-years for 32 cancer groups, 1990 to 2015: a systematic analysis for the global burden of disease study. JAMA Oncology.

[ref-33] Ghaeni FA, Amin B, Hariri AT, Meybodi NT, Hosseinzadeh H (2014). Antilithiatic effects of crocin on ethylene glycol-induced lithiasis in rats. Urolithiasis.

[ref-34] Guan X, Guan X, Dong C, Jiao Z (2020). Rho GTPases and related signaling complexes in cell migration and invasion. Experimental Cell Research.

[ref-35] Guo ZL, Li MX, Li XL, Wang P, Wang WG, Du WZ, Yang ZQ, Chen SF, Wu D, Tian XY (2021). Crocetin: a systematic review. Frontiers in Pharmacology.

[ref-36] Hashemi SA, Bathaie SZ, Mohagheghi MA (2020). Crocetin and crocin decreased cholesterol and triglyceride content of both breast cancer tumors and cell lines. Avicenna Journal of Phytomedicine.

[ref-37] Hashemzaei M, Mamoulakis C, Tsarouhas K, Georgiadis G, Lazopoulos G, Tsatsakis A, Shojaei Asrami E, Rezaee R (2020). Crocin: a fighter against inflammation and pain. Food and Chemical Toxicology.

[ref-38] He B, Tian J, Li CH, Liu Y (2010). Content variation of 4 mainly components in Fructus Gardeniae of different maturity anddifferent parts. Chinese Journal of Pharmaceutical Analysis.

[ref-39] Hire RR, Srivastava S, Davis MB, Kumar Konreddy A, Panda D (2017). Antiproliferative activity of crocin involves targeting of microtubules in breast cancer cells. Scientific Reports.

[ref-40] Hoshyar R, Bathaie SZ, Sadeghizadeh M (2013). Crocin triggers the apoptosis through increasing the Bax/Bcl-2 ratio and caspase activation in human gastric adenocarcinoma, AGS, cells. DNA and Cell Biology.

[ref-41] Hoshyar R, Khayati GR, Poorgholami M, Kaykhaii M (2016). A novel green one-step synthesis of gold nanoparticles using crocin and their anti-cancer activities. Journal of Photochemistry and Photobiology B.

[ref-42] Hoshyar R, Mollaei H (2017). A comprehensive review on anticancer mechanisms of the main carotenoid of saffron, crocin. Journal of Pharmacy and Pharmacology.

[ref-43] Huang B, Song BL, Xu C (2020). Cholesterol metabolism in cancer: mechanisms and therapeutic opportunities. Nature Metabolism.

[ref-44] Hussain MA, Abogresha NM, AbdelKader G, Hassan R, Abdelaziz EZ, Greish SM (2021). Antioxidant and anti-inflammatory effects of crocin ameliorate doxorubicin-induced nephrotoxicity in rats. Oxidative Medicine and Cellular Longevity.

[ref-45] Jahromi AS, Kargar M, Kafilzadeh F, Jamalidoust M, Moradzadeh M (2021). Crocin promotes apoptosis in human EBV-transformed B-lymphocyte *via* intrinsic pathway. Mediterranean Journal of Hematology and Infectious Diseases.

[ref-46] Jarrett AM, Lima E, Hormuth 2nd DA, McKenna MT, Feng X, Ekrut DA, Resende ACM, Brock A, Yankeelov TE (2018). Mathematical models of tumor cell proliferation: a review of the literature. Expert Review of Anticancer Therapy.

[ref-47] Javandoost A, Afshari A, Nikbakht-Jam I, Khademi M, Eslami S, Nosrati M, Foroutan-Tanha M, Sahebkar A, Tavalaie S, Ghayour-Mobarhan M, Ferns G, Hadizadeh F, Tabassi A, Mohajeri A (2017). Effect of crocin, a carotenoid from saffron, on plasma cholesteryl ester transfer protein and lipid profile in subjects with metabolic syndrome: a double blind randomized clinical trial. ARYA Atherosclerosis.

[ref-48] Jiang Z, Gu M, Liu J, Li H, Peng J, Zhang Y (2018). Anticancer activity of crocin against cervical carcinoma (HeLa cells): bioassessment and toxicity evaluation of crocin in male albino rats. Journal of Photochemistry and Photobiology B.

[ref-49] Johnson DE, O’Keefe RA, Grandis JR (2018). Targeting the IL-6/JAK/STAT3 signalling axis in cancer. Nature Reviews Clinical Oncology.

[ref-50] Joseph C, Alsaleem M, Orah N, Narasimha PL, Miligy IM, Kurozumi S, Ellis IO, Mongan NP, Green AR, Rakha EA (2020). Elevated MMP9 expression in breast cancer is a predictor of shorter patient survival. Breast Cancer Research and Treatment.

[ref-51] Kawabata K, Tung NH, Shoyama Y, Sugie S, Mori T, Tanaka T (2012). Dietary crocin inhibits colitis and colitis-associated colorectal carcinogenesis in male ICR mice. Evidence-Based Complementary and Alternative Medicine.

[ref-52] Kazemi F, Vosough I, Sepahi S, Mohajeri SA (2021). Effect of crocin *versus* fluoxetine in treatment of mild to moderate obsessive-compulsive disorder: a double blind randomized clinical trial. Human Psychopharmacology.

[ref-53] Khan I, Joshi G, Sarkar B, Nakhate KT, Ajazuddin, Mantha AK, Kumar R, Kaul A, Chaturvedi S, Mishra AK, Gupta U (2020). Doxorubicin and crocin co-delivery by polymeric nanoparticles for enhanced anticancer potential *in vitro* and *in vivo*. ACS Applied Bio Materials.

[ref-54] Khavari A, Bolhassani A, Alizadeh F, Bathaie SZ, Balaram P, Agi E, Vahabpour R (2015). Chemo-immunotherapy using saffron and its ingredients followed by E7-NT (gp96) DNA vaccine generates different anti-tumor effects against tumors expressing the E7 protein of human papillomavirus. Archives of Virology.

[ref-55] Kim B, Park B (2018). Saffron carotenoids inhibit STAT3 activation and promote apoptotic progression in IL-6-stimulated liver cancer cells. Oncology Reports.

[ref-56] Klionsky DJ, Cuervo AM, Seglen PO (2007). Methods for monitoring autophagy from yeast to human. Autophagy.

[ref-57] Kulsoom B, Shamsi TS, Afsar NA, Memon Z, Ahmed N, Hasnain SN (2018). Bax, Bcl-2, and Bax/Bcl-2 as prognostic markers in acute myeloid leukemia: are we ready for Bcl-2-directed therapy?. Cancer Management and Research.

[ref-58] Kyriakoudi A, Tsimidou MZ, O’Callaghan YC, Galvin K, O’Brien NM (2013). Changes in total and individual crocetin esters upon *in vitro* gastrointestinal digestion of saffron aqueous extracts. Journal of Agricultural and Food Chemistry.

[ref-59] Li J (2020). Saffron. Henan Agriculture.

[ref-60] Li W, He P, Huang Y, Li YF, Lu J, Li M, Kurihara H, Luo Z, Meng T, Onishi M, Ma C, Jiang L, Hu Y, Gong Q, Zhu D, Xu Y, Liu R, Liu L, Yi C, Zhu Y, Ma N, Okamoto K, Xie Z, Liu J, He RR, Feng D (2021a). Selective autophagy of intracellular organelles: recent research advances. Theranostics.

[ref-61] Li P, Ji BY, Wei J, Du XJ, Huang F (2021b). Synergistic inhibitory effects of crocin and cisplatin on human cervical cancer Hela cells. Anti-tumor Pharmacy.

[ref-62] Li CY, Lee EJ, Wu TS (2004). Antityrosinase principles and constituents of the petals of *Crocus sativus*. Journal of Natural Products.

[ref-63] Li YJ, Lei YH, Yao N, Wang CR, Hu N, Ye WC, Zhang DM, Chen ZS (2017). Autophagy and multidrug resistance in cancer. Chinese Journal of Cancer.

[ref-64] Li L, Zhang H, Jin S, Liu C (2018). Effects of crocin on inflammatory activities in human fibroblast-like synoviocytes and collagen-induced arthritis in mice. Immunologic Research.

[ref-65] Liao BL, Zhou Y, Xu QH, Tian TN, Chang N, Zhang HJ (2022). Crocetin affects the proliferation, invasion and apoptosis of gastric cancer cell line MGC-803 byregulating tubulin Saffin affects the proliferation, invasion and apoptosis of gastric cancer cellline MGC-803 by regulating tubulin. Modern Digestion & Intervention.

[ref-66] Liu BL (2016). Effect of crocin on the apoptosis of HPAC cells in human pancreatic carcinoma. Modern Medicine Journal of China.

[ref-67] Liu Z, Ding Y, Ye N, Wild C, Chen H, Zhou J (2016). Direct activation of bax protein for cancer therapy. Medicinal Research Reviews.

[ref-68] Liu D, Liu FY, Huang Y, Duan YH, Deng AH (2021). Research progress of crocin. Farm Products Processing.

[ref-69] Liu W, Sun Y, Cheng Z, Guo Y, Liu P, Wen Y (2018). Crocin exerts anti-inflammatory and anti-arthritic effects on type II collagen-induced arthritis in rats. Pharmaceutical Biology.

[ref-70] Luo Y, Cui S, Tang F, Shen C, Qi Y, Lu D, Ma L, Yang Y, Li Y, Chen R, Ri-Li GE (2017). The combination of crocin with cisplatin suppresses growth of gastric carcinoma cell line BGC-823 and promotes cell apoptosis. Pakistan Journal of Pharmaceutical Sciences.

[ref-71] Luo L, Fang K, Dan X, Gu M (2019). Crocin ameliorates hepatic steatosis through activation of AMPK signaling in db/db mice. Lipids in Health and Disease.

[ref-72] Luo Y, Yu P, Zhao J, Guo Q, Fan B, Diao Y, Jin Y, Wu J, Zhang C (2021). Inhibitory effect of crocin against gastric carcinoma *via* regulating TPM4 gene. OncoTargets and Therapy.

[ref-73] Mahdizadeh S, Karimi G, Behravan J, Arabzadeh S, Lage H, Kalalinia F (2016). Crocin suppresses multidrug resistance in MRP overexpressing ovarian cancer cell line. Daru.

[ref-74] Matthews HK, Bertoli C, De Bruin RAM (2022). Cell cycle control in cancer. Nature Reviews Molecular Cell Biology.

[ref-75] Merino Salvador M, Gómez de Cedrón M, Moreno Rubio J, Falagán Martínez S, Sánchez Martínez R, Casado E, Ramírez de Molina A, Sereno M (2017). Lipid metabolism and lung cancer. Critical Reviews in Oncology/Hematology.

[ref-76] Milani A, Basirnejad M, Shahbazi S, Bolhassani A (2017). Carotenoids: biochemistry, pharmacology and treatment. British Journal of Pharmacology.

[ref-77] Mirzaee S, Ehsan Bayatpoor M, Shahyad S, Taghi Mohammadi M, Bahari Z (2019). The protective effects of Crocin on testopathy in fat-fed and streptozotocin-treated diabetic rats: an experimental study. International Journal of Reproductive BioMedicine.

[ref-78] Mohammadi E, Mehri S, Bostan HBadie, Hosseinzadeh H (2018). Protective effect of crocin against d-galactose-induced aging in mice. Avicenna Journal of Phytomedicine.

[ref-79] Mollaei H, Safaralizadeh R, Babaei E, Abedini MR, Hoshyar R (2017). The anti-proliferative and apoptotic effects of crocin on chemosensitive and chemoresistant cervical cancer cells. Biomedicine & Pharmacotherapy.

[ref-80] Moloney JN, Cotter TG (2018). ROS signalling in the biology of cancer. Seminars in Cell & Developmental Biology.

[ref-81] Mousavi SH, Moallem SA, Mehri S, Shahsavand S, Nassirli H, Malaekeh-Nikouei B (2011). Improvement of cytotoxic and apoptogenic properties of crocin in cancer cell lines by its nanoliposomal form. Pharmaceutical Biology.

[ref-82] Mozaffari S, Ramezany Yasuj S, Motaghinejad M, Motevalian M, Kheiri R (2019). Crocin acting as a neuroprotective agent against methamphetamine-induced neurodegeneration *via* CREB-BDNF signaling pathway. Iranian Journal of Pharmaceutical Research.

[ref-83] Nasimian A, Farzaneh P, Tamanoi F, Bathaie SZ (2020). Cytosolic and mitochondrial ROS production resulted in apoptosis induction in breast cancer cells treated with Crocin: The role of FOXO3a, PTEN and AKT signaling. Biochemical Pharmacology.

[ref-84] Pachikian BD, Copine S, Suchareau M, Deldicque L (2021). Effects of saffron extract on sleep quality: a randomized double-blind controlled clinical trial. Nutrients.

[ref-85] Palassi S, Valizadeh H, Allahyari S, Zakeri-Milani P (2021). Preparation and *in vitro* characterization of enoxaparin nano-liposomes through different methods. Advanced Pharmaceutical Bulletin.

[ref-86] Pistritto G, Trisciuoglio D, Ceci C, Garufi A, D’Orazi G (2016). Apoptosis as anticancer mechanism: function and dysfunction of its modulators and targeted therapeutic strategies. Aging.

[ref-87] Puglia C, Santonocito D, Musumeci T, Cardile V, Graziano ACE, Salerno L, Raciti G, Crascì L, Panico AM, Puglisi G (2019). Nanotechnological approach to increase the antioxidant and cytotoxic efficacy of crocin and crocetin. Planta Medica.

[ref-88] Rahaiee S, Hashemi M, Shojaosadati SA, Moini S, Razavi SH (2017). Nanoparticles based on crocin loaded chitosan-alginate biopolymers: antioxidant activities, bioavailability and anticancer properties. International Journal of Biological Macromolecules.

[ref-89] Rahimi G, Shams S, Aslani MR (2022). Effects of crocin supplementation on inflammatory markers, lipid profiles, insulin and cardioprotective indices in women with PCOS: A randomized, double-blind, placebo-controlled trial. Phytotherapy Research.

[ref-90] Rahmani J, Manzari N, Thompson J, Clark CCT, Villanueva G, Varkaneh HK, Mirmiran P (2019). The effect of saffron on weight and lipid profile: a systematic review, meta-analysis, and dose–response of randomized clinical trials. Phytotherapy Research.

[ref-91] Randhawa MA, Alghamdi MS (2011). Anticancer activity of Nigella sativa (black seed)—a review. The American Journal of Chinese Medicine.

[ref-92] Rastgoo M, Hosseinzadeh H, Alavizadeh H, Abbasi A, Ayati Z, Jaafari MR (2013). Antitumor activity of PEGylated nanoliposomes containing crocin in mice bearing C26 colon carcinoma. Planta Medica.

[ref-93] Sajjadi M, Bathaie Z (2017). Comparative study on the preventive effect of saffron carotenoids, crocin and crocetin, in NMU-induced breast cancer in rats. Cell Journal.

[ref-94] Salek R, Dehghani M, Mohajeri SA, Talaei A, Fanipakdel A, Javadinia SA (2021). Amelioration of anxiety, depression, and chemotherapy related toxicity after crocin administration during chemotherapy of breast cancer: a double blind, randomized clinical trial. Phytotherapy Research.

[ref-95] Schunk SJ, Floege J, Fliser D, Speer T (2021). WNT- *β*-catenin signalling—a versatile player in kidney injury and repair. Nature Reviews Nephrology.

[ref-96] Sepahi S, Mohajeri SA, Hosseini SM, Khodaverdi E, Shoeibi N, Namdari M, Tabassi SAS (2018). Effects of crocin on diabetic maculopathy: a placebo-controlled randomized clinical trial. American Journal of Ophthalmology.

[ref-97] Sheng L, Qian Z, Zheng S, Xi L (2006). Mechanism of hypolipidemic effect of crocin in rats: crocin inhibits pancreatic lipase. European Journal of Pharmacology.

[ref-98] Shi L, Zhao S, Chen Q, Wu Y, Zhang J, Li N (2018). Crocin inhibits RANKL-induced osteoclastogenesis by regulating JNK and NF- *κ*B signaling pathways. Molecular Medicine Reports.

[ref-99] Song R, Han S, Gao H, Jiang H, Li X (2022). Crocin alleviates cognitive impairment associated with atherosclerosis *via* improving neuroinflammation in LDLR(-/-) mice fed a high-fat/cholesterol diet. Phytotherapy Research.

[ref-100] Srinivas US, Tan BWQ, Vellayappan BA, Jeyasekharan AD (2019). ROS and the DNA damage response in cancer. Redox Biology.

[ref-101] Sun Y, Xu HJ, Zhao YX, Wang LZ, Sun LR, Wang Z, Sun XF (2013). Crocin exhibits antitumor effects on human leukemia hl-60 cells *in vitro* and *in vivo*. Evidence-Based Complementary and Alternative Medicine.

[ref-102] Sung H, Ferlay J, Siegel RL, Laversanne M, Soerjomataram I, Jemal A, Bray F (2021). Global cancer statistics 2020: GLOBOCAN estimates of incidence and mortality worldwide for 36 cancers in 185 countries. CA: A Cancer Journal for Clinicians.

[ref-103] Tang Y, Yang H, Yu J, Li Z, Xu Q, Ding B, Jia G (2022). Crocin induces ROS-mediated papillary thyroid cancer cell apoptosis by modulating the miR-34a-5p/PTPN4 axis *in vitro*. Toxicology and Applied Pharmacology.

[ref-104] Teng S, Hao J, Bi H, Li C, Zhang Y, Zhang Y, Han W, Wang D (2021). The protection of crocin against ulcerative colitis and colorectal cancer *via* suppression of NF- *κ*B-mediated inflammation. Frontiers in Pharmacology.

[ref-105] Tian TD, Yue LY, Tian TL, Fan YX, Zhang XF, Ma XH (2017). Relationship between tumor lnflammation microenvironment and lmmunity along withintervening strategy of Chinese medicine. Journal of Traditional Chinese Medicine.

[ref-106] Torres MA, Raju U, Molkentine D, Riesterer O, Milas L, Ang KK (2011). AC480, formerly BMS-599626, a pan Her inhibitor, enhances radiosensitivity and radioresponse of head and neck squamous cell carcinoma cells *in vitro* and *in vivo*. Investigational New Drugs.

[ref-107] Vazifedan V, Mousavi SH, Sargolzaei J, Soleymanifard S, Fani Pakdel A (2017). Study of crocin & radiotherapy-induced cytotoxicity and apoptosis in the head and neck cancer (HN-5) cell line. Iranian Journal of Pharmaceutical Research.

[ref-108] Wang J, Ke Y, Shu T (2020). Crocin has pharmacological effects against the pathological behavior of colon cancer cells by interacting with the STAT3 signaling pathway. Experimental and Therapeutic Medicine.

[ref-109] Wang Z, Ren J, Jin N, Liu X, Li X (2020). Is crocin a potential anti-tumor candidate targeting microtubules? Computational insights from molecular docking and dynamics simulations. Frontiers in Molecular Biosciences.

[ref-110] Wu Z, Hui J (2020). Crocin reverses 1-methyl-3-nitroso-1-nitroguanidine (MNNG)-induced malignant transformation in GES-1 cells through the Nrf2/Hippo signaling pathway. Journal of Gastrointestinal Oncology.

[ref-111] Xi L, Qian Z, Xu G, Zheng S, Sun S, Wen N, Sheng L, Shi Y, Zhang Y (2007). Beneficial impact of crocetin, a carotenoid from saffron, on insulin sensitivity in fructose-fed rats. Journal of Nutritional Biochemistry.

[ref-112] Xia D (2015). Ovarian cancer HO-8910 cell apoptosis induced by crocin *in vitro*. Natural Product Communications.

[ref-113] Xiao Q, Shu R, Wu C, Tong Y, Xiong Z, Zhou J, Yu C, Xie X, Fu Z (2020). Crocin-I alleviates the depression-like behaviors probably *via* modulating microbiota-gut-brain axis in mice exposed to chronic restraint stress. Journal of Affective Disorders.

[ref-114] Xu X, Lai Y, Hua ZC (2019). Apoptosis and apoptotic body: disease message and therapeutic target potentials. Bioscience Reports.

[ref-115] Xu CG, Luo XS (2019). Crocin combined with cisplatin inhibits proliferation and apoptosis of gastric cancer cells by inhibiting ERK signaling pathway. Journal of Shanxi Medical University.

[ref-116] Xu Q, Yu J, Jia G, Li Z, Xiong H (2022). Crocin attenuates NF- *κ*B-mediated inflammation and proliferation in breast cancer cells by down-regulating PRKCQ. Cytokine.

[ref-117] Xu HJ, Zhang KP, Zhong R, Zhao YX, Li XR, Lu Y, Song AQ, Pang XY, Sun LR (2012). Influence of crocin on proliferation *in vitro* and function of dendritic cells derived from bone marrow of children with acute leukemia. Journal of Experimental Hematology.

[ref-118] Yamazaki T, Bravo-San Pedro JM, Galluzzi L, Kroemer G, Pietrocola F (2021). Autophagy in the cancer-immunity dialogue. Advanced Drug Delivery Reviews.

[ref-119] Yang H, Yu J, Wang L, Ding D, Zhang L, Chu C, Chen Q, Xu Z, Zou Q, Liu X (2014). miR-320a is an independent prognostic biomarker for invasive breast cancer. Oncology Letters.

[ref-120] Yarijani ZM, Pourmotabbed A, Pourmotabbed T, Najafi H (2017). Crocin has anti-inflammatory and protective effects in ischemia-reperfusion induced renal injuries. Iranian Journal of Basic Medical Sciences.

[ref-121] Yarla N, Madka V, Rao C (2022). Targeting triglyceride metabolism for colorectal cancer prevention and therapy. Current Drug Targets.

[ref-122] Yi M, Li J, Chen S, Cai J, Ban Y, Peng Q, Zhou Y, Zeng Z, Peng S, Li X, Xiong W, Li G, Xiang B (2018). Emerging role of lipid metabolism alterations in Cancer stem cells. Journal of Experimental & Clinical Cancer Research.

[ref-123] Yin Q, Xiong H (2022). Chemotherapy-induced nephrotoxicity was improved by crocin in mouse model. European Journal of Histochemistry.

[ref-124] Yu H, Lin L, Zhang Z, Zhang H, Hu H (2020). Targeting NF- *κ*B pathway for the therapy of diseases: mechanism and clinical study. Signal Transduction and Targeted Therapy.

[ref-125] Yu F, Yu C, Li F, Zuo Y, Wang Y, Yao L, Wu C, Wang C, Ye L (2021). Wnt/ *β*-catenin signaling in cancers and targeted therapies. Signal Transduction and Targeted Therapy.

[ref-126] Zarei Jaliani H, Riazi GH, Ghaffari SM, Karima O, Rahmani A (2013). The effect of the *Crocus sativus* L. Carotenoid, crocin, on the polymerization of microtubules, *in vitro*. Iranian Journal of Basic Medical Sciences.

[ref-127] Zeng A, Hua H, Liu L, Zhao J (2019). Betulinic acid induces apoptosis and inhibits metastasis of human colorectal cancer cells *in vitro* and *in vivo*. Bioorganic & Medicinal Chemistry.

[ref-128] Zeng C, Li H, Fan Z, Zhong L, Guo Z, Guo Y, Xi Y (2016). Crocin-elicited autophagy rescues myocardial ischemia/reperfusion injury *via* paradoxical mechanisms. The American Journal of Chinese Medicine.

[ref-129] Zhang Y, Brekken RA (2022). Direct and indirect regulation of the tumor immune microenvironment by VEGF. Journal of Leukocyte Biology.

[ref-130] Zhang H, Lin J, Shen Y, Pan J, Wang C, Cheng L (2022b). Protective effect of crocin on immune checkpoint inhibitors-related myocarditis through inhibiting NLRP3 mediated pyroptosis in cardiomyocytes *via* NF- *κ*B pathway. Journal of Inflammation Research.

[ref-131] Zhang F, Liu P, He Z, Zhang L, He X, Liu F, Qi J (2022a). Crocin ameliorates atherosclerosis by promoting the reverse cholesterol transport and inhibiting the foam cell formation *via* regulating PPAR *γ*/LXR- *α*. Cell Cycle.

[ref-132] Zhang C, Liu J, Xu D, Zhang T, Hu W, Feng Z (2020a). Gain-of-function mutant p53 in cancer progression and therapy. Journal of Molecular Cell Biology.

[ref-133] Zhang K, Wang L, Si S, Sun Y, Pei W, Ming Y, Sun L (2018). Crocin improves the proliferation and cytotoxic function of T cells in children with acute lymphoblastic leukemia. Biomedicine & Pharmacotherapy.

[ref-134] Zhang J, Yang S, Wang K, Huang Y, Yang N, Yang Z, Zheng Z, Wang Y (2020b). Crocin induces autophagic cell death and inhibits cell invasion of cervical cancer SiHa cells through activation of PI3K/AKT. Annals of Translational Medicine.

[ref-135] Zhang Y, Zhu M, Mohan SKrishna, Hao Z (2021). Crocin treatment promotes the oxidative stress and apoptosis in human thyroid cancer cells FTC-133 through the inhibition of STAT/JAK signaling pathway. Journal of Biochemical and Molecular Toxicology.

[ref-136] Zhong K, Qian C, Lyu R, Wang X, Hu Z, Yu J, Ma J, Ye Y (2022). Anti-epileptic effect of crocin on experimental temporal lobe epilepsy in mice. Frontiers in Pharmacology.

[ref-137] Zhou Y, Xu Q, Shang J, Lu L, Chen G (2019). Crocin inhibits the migration, invasion, and epithelial-mesenchymal transition of gastric cancer cells *via* miR-320/KLF5/HIF-1 *α* signaling. Journal of Cellular Physiology.

